# Efficacy of pancreatic enzyme replacement therapy in chronic pancreatitis: systematic review and meta-analysis

**DOI:** 10.1136/gutjnl-2016-312529

**Published:** 2016-12-09

**Authors:** Daniel de la Iglesia-García, Wei Huang, Peter Szatmary, Iria Baston-Rey, Jaime Gonzalez-Lopez, Guillermo Prada-Ramallal, Rajarshi Mukherjee, Quentin M Nunes, J Enrique Domínguez-Muñoz, Robert Sutton, NIHR Pancreas Biomedical Research Unit Patient Advisory Group

**Affiliations:** 1 NIHR Liverpool Pancreas Biomedical Research Unit, Royal Liverpool University Hospital, University of Liverpool, Liverpool, UK; 2 Department of Gastroenterology and Hepatology, University Hospital of Santiago de Compostela, Compostela, Spain; 3 Department of Integrated Traditional Chinese and Western Medicine, Sichuan Provincial Pancreatitis Centre, West China Hospital, Sichuan University, Chengdu, China; 4 Department of Pharmacy, University Hospital of Santiago de Compostela, Compostela, Spain; 5 Department of Preventive Medicine and Public Health, University of Santiago de Compostela, Compostela, Spain

**Keywords:** EXOCRINE PANCREATIC FUNCTION, NUTRIENT ABSORPTION, PANCREATIC ENZYMES, PANCREATITIS

## Abstract

**Objective:**

The benefits of pancreatic enzyme replacement therapy (PERT) in chronic pancreatitis (CP) are inadequately defined. We have undertaken a systematic review and meta-analysis of randomised controlled trials of PERT to determine the efficacy of PERT in exocrine pancreatic insufficiency (EPI) from CP.

**Design:**

Major databases were searched from 1966 to 2015 inclusive. The primary outcome was coefficient of fat absorption (CFA). Effects of PERT versus baseline and versus placebo, and of different doses, formulations and schedules were determined.

**Results:**

A total of 17 studies (511 patients with CP) were included and assessed qualitatively (Jadad score). Quantitative data were synthesised from 14 studies. PERT improved CFA compared with baseline (83.7±6.0 vs 63.1±15.0, p<0.00001; I^2^=89%) and placebo (83.2±5.5 vs 67.4±7.0, p=0.0001; I^2^=86%). PERT improved coefficient of nitrogen absorption, reduced faecal fat excretion, faecal nitrogen excretion, faecal weight and abdominal pain, without significant adverse events. Follow-up studies demonstrated that PERT increased serum nutritional parameters, improved GI symptoms and quality of life without significant adverse events. High-dose or enteric-coated enzymes showed a trend to greater effectiveness than low-dose or non-coated comparisons, respectively. Subgroup, sensitive and meta-regression analyses revealed that sample size, CP diagnostic criteria, study design and enzyme dose contributed to heterogeneity; data on health inequalities were lacking.

**Conclusions:**

PERT is indicated to correct EPI and malnutrition in CP and may be improved by higher doses, enteric coating, administration during food and acid suppression. Further studies are required to determine optimal regimens, the impact of health inequalities and long-term effects on nutrition.

**Significance of this study**

**What is already known on this subject?**

- Chronic pancreatitis (CP) is a major health problem associated with health inequalities, causing intractable abdominal pain, peripancreatic complications, exocrine pancreatic insufficiency (EPI), long-term malnutrition and type 3c diabetes mellitus.
- Pancreatic enzyme replacement therapy (PERT) is administered by some for EPI in CP but the benefits remain to be confirmed.
- No meta-analysis has been conducted previously of randomised clinical trials assessing PERT for EPI in CP, while a previous Cochrane review of PERT in CP was inconclusive.

**What are the new findings?**

- This meta-analysis shows that PERT improves the coefficients of fat and nitrogen absorption versus baseline and versus placebo, reducing faecal fat excretion, faecal nitrogen excretion, faecal weight and abdominal pain without significant adverse events. Follow-up studies have found that PERT increases serum nutritional parameters, improves GI symptoms and quality of life without significant adverse events.
- Although there was significant heterogeneity between trials, subgroup analyses did not alter the findings, and exclusion of trials with small sample sizes (<40) or without imaging and/or histology to establish CP significantly reduced the heterogeneity. Data on health inequalities were sparse but for those on CP aetiology attributed to alcohol excess.

**How might it impact on clinical practice in the foreseeable future?**

- This systematic review and meta-analysis of 17 randomised trials of PERT for EPI in CP demonstrates the efficacy of PERT for correcting malnutrition in CP. PERT may be optimised by higher doses, enteric coating, ingestion during food and acid suppression.
- Further studies are needed to determine optimal methods to address the impact of health inequalities on PERT for EPI in CP.

## Introduction

Chronic pancreatitis (CP) is a progressive fibro-inflammatory disorder with sustained destruction of acinar, ductal and islet cells.^1^ CP has an annual incidence of 4–12 per 100 000,^2^ increasing worldwide.^3^ Aetiologies include environmental toxins (prolonged, heavy alcohol exposure and cigarette smoking), hyperlipidaemia, single and/or multiple genetic mutations (*PRSS1, SPINK1, CTRC, CASR, CFTR, CLDN2* and *CPA1*) and autoimmune disease.^3^ ^4^ CP is generally progressive, with marked variation in abdominal pain and GI symptoms. These greatly impair quality of life (QoL),^5^ while exocrine pancreatic insufficiency (EPI) and diabetes mellitus (DM) contribute to the low median survival of 15–20 years from diagnosis.^3^ ^6^ The prevalence of CP has been estimated at circa 50 per 100 000 but this is a significant underestimate in view of incidence and median survival, which suggests 100–200 per 100 000;^3^ similarly, EPI is probably underestimated.^3^ Not surprisingly the management of CP and its complications is resource intensive;^7^ were the management of EPI and DM from CP to be improved and complications reduced, these resources would be better spent.

EPI, characterised by inadequate pancreatic secretion of digestive enzymes and bicarbonate, is one of the most significant complications of CP affecting >50% of diagnosed patients,^8^ resulting in compromised digestion, absorption and metabolism of nutrients. Severe EPI tends to develop between 5 and 10 years following an initial diagnosis of CP^1^ and can complicate acute necrotising pancreatitis, cystic fibrosis, DM, pancreatic cancer and following surgery to bypass or resect the duodenum and/or pancreas.^9^ EPI from CP reduces absorption of fat including essential fatty acids, fat-soluble vitamins A, D, E and K, calcium, magnesium, zinc, thiamine and folic acid.^10^ EPI from CP is frequently experienced as diarrhoea, abdominal discomfort and/or pain, weight loss and grossly as steatorrhoea (strictly, >7 g fat in stool/24 hours).^1^ Intermediate and long-term malnutrition from EPI increases the incidence of osteopenia/osteoporosis,^11^ low-trauma fractures,^12^ cardiovascular diseases^13^ and infections.^6^ These complications develop on a background of health inequalities, differences between people's health as a result of social, geographical or other factors, which are associated with less favourable outcomes from CP; foremost is malnutrition.^14^ Rates of alcoholism and smoking also have significant socioeconomic gradients, as do osteopenia/osteoporosis, DM and cardiovascular diseases, making health inequality an independent risk factor for the initiation and progression of CP.^4^

Pancreatic lipase is the principal enzyme in fat digestion accounting for 90% of total lipase activity.^9^ Typically steatorrhoea is unlikely until pancreatic lipase output falls to <10% of normal,^15^ although subclinical EPI exists in many individuals with CP.^16^ Large-scale Northern European studies suggest that the majority of patients with EPI secondary to CP^17^ or pancreatic surgery^18^ are undertreated, likely because of insufficient use of pancreatic enzyme replacement therapy (PERT), suboptimal scheduling in relation to meals or insufficient control of gastric acid output. The European Society for Parenteral and Enteral Nutrition 2006 Guidelines^19^ suggest improvement of steatorrhoea and maintenance of bodyweight as primary markers of treatment success. Such end points, however, do not detect subtler signs of malnutrition; nor do these guidelines suggest formal measures to assess improvement of steatorrhoea. Furthermore, these end points are unsuitable as primary outcome measures for studies of PERT for EPI, particularly short-term studies. For these, accurate measures that detect alterations and/or improvements in absorption are necessary.^1^ ^3^ ^9^ ^16^ In practice, licensed enzyme replacement therapies differ greatly: granules, tablets, microspheres, minimicrospheres or capsules with enteric coating make comparisons challenging. While the European Medicines Agency has delegated regulation of PERT to national authorities, the US Food and Drug Administration (FDA) addressed these differences in 2004 (updated in 2006),^20^ issuing requirements for new drug applications, specifying amount, stability and efficacy. To date, six enzymes have FDA approval: Creon, Pancreaze, Pertzye, Ultrase, Viokase and Zenpep, all of porcine origin.

Questions remain as to the indications for and efficacy of PERT, including methods of assessment, formulation, dose, administration and use of acid suppression. Shafiq *et al* 21 conducted a meta-analysis of PERT for CP regardless of the presence or absence of EPI. Data for weight loss and faecal fat were combined from only two studies; the authors inferred that the evidence for any beneficial role of PERT in CP was inconclusive. Waljee *et al* 22 and Taylor *et al* 23 combined trials in cystic fibrosis and CP, concluding that PERT improves but does not normalise the coefficient of fat absorption (CFA) in EPI; these two systematic reviews included a total of only three placebo-controlled trials of PERT in CP, and no quantitative data were combined to determine outcomes. Three further placebo-controlled trials of PERT in CP have since been conducted, two of which were followed by open-label extensions. We have therefore conducted a systematic review of 17 randomised controlled trials (RCTs) with meta-analysis of 14 to determine whether PERT is indicated for EPI in CP, the impact of this treatment and factors contributing to optimisation of PERT. Our study is strengthened by meta-analysis of PERT versus baseline, PERT versus placebo and PERT versus PERT to provide a more rigorous evaluation, comparing CFA, coefficient of nitrogen absorption (CNA), faecal fat excretion (FFE), faecal nitrogen excretion (FNE), faecal weight, abdominal pain and GI symptoms, bodyweight, QoL and adverse events.

## Methods

### Search strategy

All studies of PERT for EPI from CP were sought in Medline (PubMed), Embase, Scopus, Science Citation Index Expanded and the Cochrane Central Register of Controlled Trials between January 1966 (the first PERT study) and December 2015. The following search terms were used in all possible combinations: Chronic adj3 pancreatitis, exocrine pancreatic insufficiency, pancrea$ insufficien$; Randomized (or randomised) controlled trial.pt., controlled clinical trial. pt, randomized.ab.placebo.ab. drug therapy.fs, randomly.ab.trial.ab.group.ab; Enzymes.tw,(enzyme$ adj1 (pancrea$ or replace$ or supplement$)).tw.(pancreatin or pancrease or pancrealipase or ultrase or cotazym or creon or kreon or theraclec or encron or protilase or lipase or hydrolase or exolipase or trigly-creidase or ALTU-135).tw; English.lg. A manual reference search of reviews and conference abstracts (2006–2015) was also undertaken.

Studies meeting all the following criteria were included: (1) in English peer-reviewed journals; (2) prospective, randomised design, investigating efficacy and safety of PERT in EPI from CP in adults (age ≥18 years; including patients who had pancreatic resection for CP but not other indications); (3) reporting clinical outcomes of interest; and (4) only the most recent study of multiple overlapping patient populations from the same institution unless a prior study had higher quality. Abstracts, case reports, letters, expert opinions, editorials, reviews and non-RCTs were excluded.

Preferred Reporting Items for Systematic Reviews and Meta-analyses (PRISMA) criteria^24^ were followed. Two authors (DdlI-G and WH) independently scrutinised all identified studies and reached consensus for final inclusion, with adjudication (PS) when there was disagreement.

### Data extraction

Two authors (DdlI-G and WH) extracted data independently using predefined standardised forms. These captured study design, quality assessment (below), baseline characteristics, health equality indicators (ethnicity, place of residence, socioeconomic background, employment/insurance status, profession, alcohol use and cigarette smoking), diagnostic criteria for CP and EPI, exclusion criteria, trial process, details of PERT and outcomes of interest.

### Outcomes of interest

CFA has been used most commonly as the primary end point to assess the efficacy of PERT on EPI due to CP and cystic fibrosis in RCTs, more informative than the presence or absence of steatorrhoea.^22^ ^23^ We have therefore used CFA as the primary outcome measure, calculated from fat intake (∼100 g/day of dietary fat) and excretion (from 72 hours faecal collection) using the following equation:
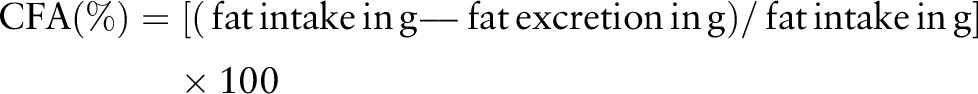



CNA has been used to measure the effect of PERT on protein absorption in EPI, ignored in previous systematic reviews;^21–23^ CNA was calculated as follows:
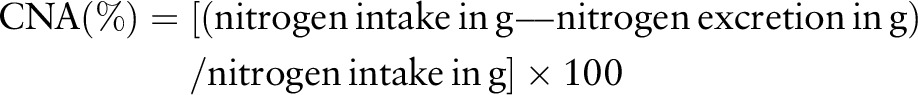



Secondary outcomes included CNA, FFE, FNE, faecal weight, faecal consistency (formed/normal or soft/watery), faecal frequency (stools per day), flatulence (none/mild/moderate/severe), abdominal pain (none/mild/moderate/severe) and adverse events. When available, serum nutritional markers, diarrhoea, weight loss/gain and QoL were included.

### Quality assessment

Two authors (DdlI-G, WH) scored each included study using the Jadad system^25^ that assesses randomisation (0 or 1), double-blinding (0, 1 or 2), recording of dropouts and/or withdrawals (0 or 1) and allocation concealment (0 or 1), with a score ≥3 indicative of high quality.

### Statistical analysis

Means and SDs of continuous variable were used for meta-analysis, estimated^26^ when medians were given. If CFA and CNA were not available, these were calculated as described. If different protocols of PERT were used in any single study, average daily lipase dose was calculated in United States Pharmacopoeia (USP) units. Meta-analyses compared PERT versus baseline, PERT versus placebo or PERT versus PERT. Forest plots were generated using Review Manager V.5.3 software (Cochrane Collaboration, Oxford, UK). Continuous variables were expressed as weighted mean differences (WMD) and categorical variables as ORs with 95% CIs. A random-effects model^27^ was employed to ensure conservative estimates in meta-analyses. Heterogeneity was evaluated using χ^2^ with p<0.1 considered significant. Statistical heterogeneity was assessed using I^2^ values with cut-offs of 25%, 50% and 75% to indicate low, moderate and high heterogeneity, respectively.^28^ Subgroup analyses examined studies of high quality; parallel, multicentre design; samples ≥40 and Western populations. Sensitivity analyses were conducted by diagnostic criteria and inclusion of pancreatic surgery.

Meta-regression analyses assessed influence of age, gender, study design, study quality, lipase dose and publication year on summary estimates using Stata SE V.13 Software (StataCorp, Texas, USA); p<0.05 was considered significant. Publication bias was assessed using funnel plots,^29^ and p values generated from both CFA and FFE as per Begg and Mazumdar^30^ and Egger *et al*;^31^ p<0.10 was considered significant.

### Patient and public involvement

The research design, methods, results and their interpretation were reviewed by the NIHR Liverpool Pancreas Biomedical Research Unit Patient Advisory Group, and modifications made accordingly. The group is comprised of patients with a history of acute pancreatitis, CP or pancreatic cancer, or their carers, or interested members of the public.

## Results

### Design and quality assessment of included studies

**Figure 1:**
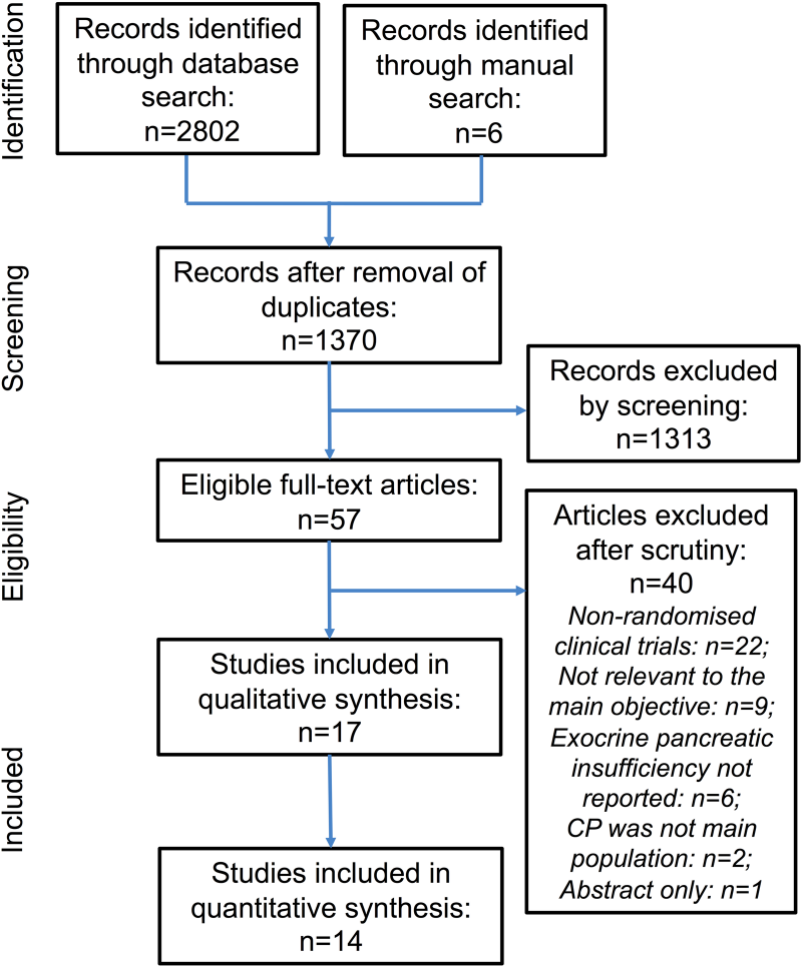
Preferred Reporting Items for Systematic Reviews and Meta-analyses flow chart of study selection process. CP, chronic pancreatitis.

The PRISMA flow diagram is shown in figure 1; 17 studies^32–48^ were included. Study design and quality assessment are shown in table 1. There were four^32^ ^33^ ^40^ ^45^ conducted in the USA, nine^34–39^ ^41^ ^43^ ^44^ in Europe, two^46^ ^47^ in the USA and Europe, one^42^ in South Africa and one^48^ in India. Twelve^32–37^ ^39–41^ ^43^ ^44^ ^47^ had cross-over designs (two^41^ ^47^ multicentre); five^38^ ^42^ ^45^ ^46^ ^48^ had parallel designs (three^45^ ^46^ ^48^ multicentre). Five^41^ ^43^ ^46–48^ reported sample size calculations, four^41^ ^46–48^ using change of CFA values and one^43^ cumulative ^13^CO_2_ recovery rate. Detailed Jadad scoring is shown in online supplementary table S1. All seven high-quality studies^35^ ^38^ ^41^ ^45–48^ were double-blinded.

**Table 1:** Design and quality assessment of included studies

Study	Year	Country	Study period	Design	Single or multicentre	Type*	No. of groups	Sample size calculation	Blinding method	Jadad score
Graham^32^	1979	USA	NR	Cross-over	Single	PERT vs PERT	5	No	NR	1
Dutta *et al* 33	1983	USA	NR	Cross-over	Single	PERT vs PERT	3	No	NR	1
Lankisch *et al* 34	1986	Germany	NR	Cross-over	Single	PERT vs PERT	3	No	NR	1
Halgreen *et al* 35	1986	Denmark	NR	Cross-over	Single	PERT vs placebo	2	No	Double	3
Gouerou *et al* 36	1989	France	NR	Cross-over	Single	PERT vs PERT	2	No	NR	2
Jørgensen *et al* 37	1991	Denmark	NR	Cross-over	Single	PERT vs PERT	3	No	NR	1
Paris^38^	1993	France	June 1986 to June 1987	Parallel	Single	PERT vs placebo	2	No	Double	3
Delhaye *et al* 39	1996	Belgium	March 1993 to May 1994	Cross-over	Single	PERT vs PERT	4	No	NR	2
Opekun Jr *et al* 40	1997	USA	NR	Cross-over	Single	PERT vs PERT vs placebo	4	No	Single	1
Halm *et al* 41	1999	Germany	NR	Cross-over	Multicentre	PERT vs PERT	2	Yes	Double	4
O’Keefe *et al* 42	2001	South Africa	NR	Parallel	Single	PERT vs placebo	2	No	NR	2
Domínguez-Muñoz *et al* 43	2005	Spain	NR	Cross-over	Single	PERT vs PERT	3	Yes	Not possible	2
Vecht *et al* 44	2006	Netherlands	NR	Cross-over	Single	PERT vs PERT	2	No	Double	2
Safdi *et al* 45	2006	USA	NR	Parallel	Multicentre	PERT vs placebo	2	No	Double	3
Whitcomb *et al* 46	2010	USA/Europe	April 2007 to August 2008	Parallel	Multicentre	PERT vs placebo	2	Yes	Double	5
Toskes *et al* 47	2011	USA/Europe	January 2008 to March 2009	Cross-over	Multicentre	PERT vs PERT	2	Yes	Double	3
Thorat *et al* 48	2012	India	June 2008 to May 2010	Parallel	Multicentre	PERT vs placebo	2	Yes	Double	5

*Refers to comparisons of different types of PERT, different doses of the same PERT.

NR, not reported; PERT, pancreatic enzyme replacement therapy.

10.1136/gutjnl-2016-312529.supp1 supplementary table

### Baseline parameters of patients with CP

These are shown in online supplementary table S2; 511 patients with CP were enrolled and 477 analysed; median age was 50 years. Gender was reported in most studies (pooled 76.4% males). Ethnicity was reported in only four.^45–48^ None reported residence data, smoking, socioeconomic status or profession. Five^41^ ^42^ ^46–48^ reported body mass index. Nine^32^ ^33^ ^35^ ^36^ ^39^ ^40^ ^42^ ^44^ ^48^ included data on coexisting DM (pooled frequency 49.5%). Patients in seven studies^32^ ^35^ ^37^ ^40–42^ ^45^ did not have pancreatic surgery recorded, while the remaining studies included such patients. Ten studies^32–36^ ^38–40^ ^42^ ^44^ reported aetiology, with alcohol excess pooled at 89.1%.

10.1136/gutjnl-2016-312529.supp2 supplementary table

### Inclusion and exclusion criteria

The diagnostic criteria for CP and EPI are shown in table 2. CP was diagnosed by imaging and/or histology in six studies,^33^ ^36^ ^39^ ^46–48^ imaging and/or history in seven^35^ ^37^ ^38^ ^41–44^ and history, abnormal secretin-pancreatozymin test and/or histology in one.^34^ The diagnostic criteria in the remaining three studies^32^ ^40^ ^45^ were unclear. EPI was defined by FFE >7–8 g/day in five,^33^ ^35^ ^36^ ^41^ ^43^ by FFE >10–15 g/day and/or CFA ≤80% in nine and by faecal elastase ≤100 µg/g in one study.^47^ One study^32^ did not report criteria for EPI and 1^40^ included CP patients with documented symptomatic steatorrhoea. Ten^36^ ^38^ ^39^ ^41–43^ ^45–48^ reported detailed exclusion criteria.

**Table 2:** Diagnostic criteria and details of trial process

Study	Criteria for chronic pancreatitis	Criteria for exocrine pancreatic insufficiency before randomisation	Run-in (washout) phase	Treatment phase	Fat in diet/day (g)	Faecal dye	Controlled timing of faecal fat collection	Adverse events
Graham^32^	NR	NR	NR	6 days for each of the four PERT preparations	100	NR	3-day equilibration followed by 3-day collection	NR
Dutta *et al* 33	Radiological evidence of pancreatic calcifications or multiple strictures in MPD, or histological evidence	Normal d-xylose absorption; marked abnormal secretin test; FFE >7 g/day on a 100 g/day fat intake	NR	3 days for each of the two PERT preparations	100	NR	3-day equilibration followed by 3-day collection	NR
Lankish *et al* 34	Typical disease history and/or histology	Abnormal secretin-pancretozymin test; FFE >15 g/day	3 days	5 days for each of the three PERT preparations	100	NR	2-day equilibration followed by 3-day collection	NR
Halgreen *et al* 35	Imaging indicative of pancreatic calcification, previous acute attacks and/or typical abnormalities by ERCP	Meal stimulated duodenal lipase <50 kU/L and FFE >8 g/day	14 days	14 days for PERT or placebo then vice versa	100	NR	2-day equilibration followed by 3-day collection	NR
Gouerou *et al* 36	Imaging indicative of pancreatic calcification, ERCP abnormalities or other histological signs	FFE >8 g/day	10 days	21 days each for either two PERT preparations then vice versa	NR	NR	3 days before treatment and 3 days at the end of each treatment period	PERT1: 11.4%; PERT2: 11.4%
Jørgensen *et al* 37	Pancreatic calcifications on US, ERCP, CT and/or anatomy abnormalities in laparotomy	Meal stimulated duodenal enzyme activity below 10% and FFE 15 g/day on free diet	7 days	7 days for each of the two PERT preparations	NR	Yes, brilliant blue	3 days before treatment and after 7 days of each treatment	NR
Paris^38^	Radiology of preoperative confirmation	FFE >10 g/day	7–9 days placebo followed by 5 days	8-day PERT or placebo	>100	NR	4-day equilibration followed by 3-day collection	PERT: 10.3%; placebo: 9.1%
Delhaye *et al* 39	Pancreatic calcifications, typical abnormalities in ERCP, or histology	FFE >10 g/day	NR	14 days for each of the four PERT preparations followed by 5 days of standard diet period in between	>100	NR	14-day equilibration followed by 3- day collection	NR
Opekun Jr *et al* 40	NR	Documented symptomatic steatorrhoea	6- day placebo	6 days for each of the three PERT preparations and a placebo with 2-day washout period in between	100	NR	3-day equilibration followed by 3-day collection	NR
Halm *et al* 41	US, CT, ERCP or X-ray indicative of pancreatic calcification and <6 acute attacks	FFE >7.5 g/day	7-day placebo followed by 7-day PERT MS	14 days of each two PERT MS and MMS with a 7-day washout period in between	70–80	NR	4-day equilibration followed by 3-day collection in placebo period, 11-day equilibration followed by 3-day collection in PERT period	PERT MS: 21.7%; PERT MMS: 17.4%
O'Keefe *et al* 42	Typical signs of chronic pancreatitis in CT, US, ERCP or pancreatic calcification in X-ray	FFE >10 g/day	7-day placebo followed by 7-day PERT	14-day PERT or placebo	∼100	NR	4-day equilibration followed by 3-day collection in placebo period, 11-day equilibration followed by 3-day collection in PERT period	NR
Domínguez- Muñoz *et al* 43	Severe chronic pancreatitis diagnosed by MRI, CT and/or EUS (Cambridge and Wiersema criteria)	FFE >7 g/day	>5 days	7 days for each of the three PERT preparations (consecutive)	92	NR	5-day equilibration followed by 3- day collection	NR
Vecht *et al* 44	Clinical history, alterations of pancreatic morphology in CT, ERCP (Cambridge score)	FFE >10 g/day	15 days	15 days each for either high or low dose of PERT preparation then vice versa	NR	NR	3-day collection	NR
Safdi *et al* 45	Documented chronic pancreatitis	CFA <80% and/or FFE >10 g/day in run-in phase; study compliance	14-day placebo	14-day PERT or placebo	>100	NR	11-day equilibration followed by 3-day collection	PERT: 23.1%; placebo: 35.7%
Whitcomb *et al* 46	Medical history and imaging indicative calcification and/or histology	CFA <80% and/or total faecal fat content ≥10 g/day; study compliance	5-day placebo	7-day PERT or placebo	>100	Yes, indigo carmine	2-day equilibration followed by 3-day collection in run-in phase, 4-day equilibration followed by 3-day collection in PERT period	PERT: 20.0%; placebo: 20.7%
Toskes *et al* 47	Medical history and one of the criteria: ERCP Cambridge 4, CT with dilated MPD, atrophy, calcification, US, EUS with more than five criteria; partial or distal pancreatectomy not due to cancer	Faecal elastase ≤100 µg/g	7–9-day placebo	∼8 days each for either high or low dose of PERT preparations then vice versa	100	Yes, indigo carmine	4-day equilibration followed by 3-day collection in run-in phase, 6-day equilibration followed by 3-day collection in each PERT period	Low-dose PERT: 39.2%; high-dose PERT: 41.3%; placebo: 42.7%
Thorat *et al* 48	Imaging indicative calcifications or MPD dilatation and/or histology	CFA ≤80%	7-day followed by 7-day PERT	7-day PERT or placebo	100	NR	4-day equilibration followed by 3-day collection	PERT: 35.3%; placebo: 25.0%

CFA, coefficient of fat absorption; ERCP, endoscopic retrograde cholangiopancreatography; EUS, endoscopic ultrasonography; FFE, faecal fat absorption; MMS, minimicrospheres; MPD, main pancreatic duct; MS, microspheres; NR, not reported; PERT, pancreatic enzyme replacement therapy; US, ultrasonography.

### Details of trial process

The trial process and adverse events are shown in table 2. RCTs of PERT typically had a run-in phase to scrutinise and stabilise eligible participants before treatment initiation. During run-in or washout, PERT was stopped. Six studies^34–37^ ^43^ ^44^ reported a 3–15-day no-treatment run-in, while five^38^ ^40^ ^45–47^ had a 5–14-day period for placebo administration before commencing the trial. Three studies^41^ ^42^ ^48^ reported a 14-day run-in with the last seven days before randomisation treated by PERT. The remaining three studies^32^ ^33^ ^39^ did not report a run-in phase. During treatment, PERT or placebo was used with or without a washout in between switching treatments. Daily fat intake was recorded in the majority (normally 100 g/day), but not in three.^36^ ^37^ ^44^ Only three^37^ ^46^ ^47^ reported use of a faecal dye. The equilibration time was normally 2–5 days before a 3-day period of faecal fat collection. Adverse events were reported in six,^36^ ^41^ ^45–48^ with an incidence of 11.4–42.7%.

RCT study duration ranged from several days to 2 months; none assessed long-term effects of PERT, although two open-label extension studies examined the nutritional impact of PERT over 6 months^49^ and 12 months.^50^

### Composition and administration schedule of PERT

**Figure 2:**
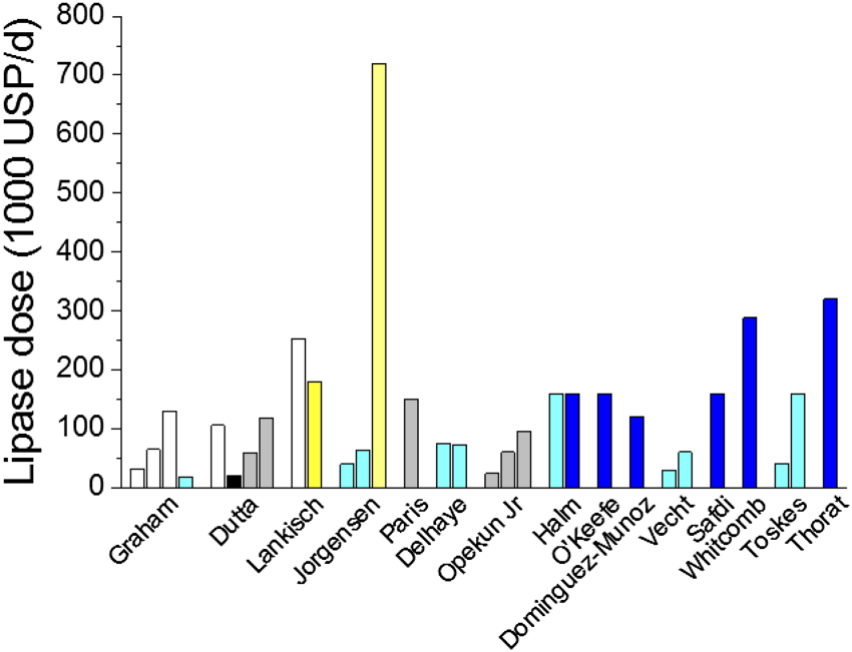
Daily lipase dose of pancreatic enzymes in the reported studies. Black, non-EC microspheres; blue, minimicrospheres; cyan, EC microspheres; EC, enteric-coated; grey, EC microtablets; USP, United States Pharmacopoeia; white, non-EC microtablets; yellow, EC granules.

Details of PERT composition and administration schedules are shown in online supplementary table S3. Converted pancreatic lipase doses in USP units are shown in figure 2. The source of pancreatic enzymes and conversion factors is shown in online supplementary table S4. PERT formulations were granules, microtablets, microspheres and minimicrospheres with or without enteric coating. Four studies^32–34^ ^36^ included non-coated and enteric-coated enzymes. Two studies^46^ ^47^ used delayed release enteric-coated minimicrospheres (Creon 12000) or microspheres (Zenpep).

10.1136/gutjnl-2016-312529.supp3 supplementary table

10.1136/gutjnl-2016-312529.supp4 supplementary table

Delhaye's study^39^ found no significant differences in the efficacy of Pancrease HL and Creon 3 (both enteric-coated microspheres). Halm's study^41^ showed the primary outcome CFA and adverse events to be similar for Creon 10000 microspheres and minimicrospheres. Vecht's study^44^ found low-dose (lipase 10 000 USP units tds) improved fat absorption and reduced symptoms compared with normal dose (lipase 20 000 USP units tds) Pancrease when combined with strong acid inhibition (omeprazole 60 mg). Toskes's study^47^ showed low-dose (lipase 7×5000 USP units/day) and high-dose (lipase 7×20 000 USP units/day) Zenpep without acid inhibition both significantly improved CFA, CNA, bodyweight and body mass index. Subgroup analysis, however, revealed that a higher dose of PERT may be needed for more severe EPI.

Domínguez-Muñoz's study^43^ suggested that PERT administration during or after meals may be more appropriate than before meals. In all other studies, PERT was taken before or with meals or snacks at various different doses. The use of proton pump or H_2_ inhibitors was reported or allowed in five^34^ ^39^ ^44^ ^46^ ^48^ with only one^39^ comparing PERT with versus PERT without omeprazole; in this last study, omeprazole improved fat digestion but compromised protein digestion.

### Meta-analysis results

In total, 14 of 17 included RCTs^32–38^ ^40^ ^42^ ^44–48^ had data on predefined clinical outcomes of interest suitable for quantitative comparison of PERT versus baseline, versus placebo and versus PERT.

### PERT versus baseline

**Figure 3:**
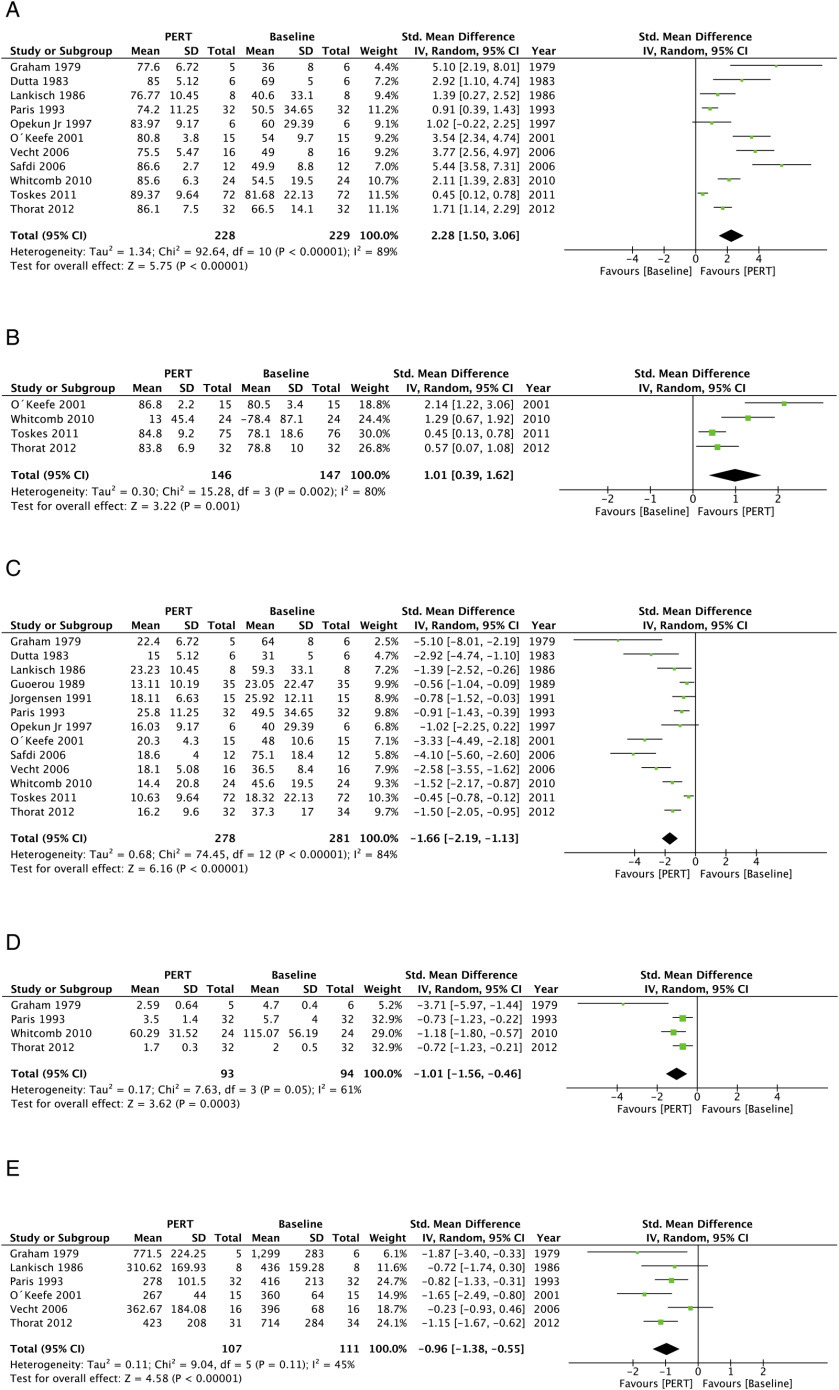
The pooled clinical outcomes of pancreatic enzyme replacement therapy (PERT) versus baseline. (A) coefficient of fat absorption (CFA), (B) coefficient of nitrogen absorption (CNA), (C) faecal fat excretion (FFE), (D) faecal nitrogen excretion (FNE) and (E) faecal weight.

The clinical outcomes of PERT versus baseline are presented in figure 3 and summarised in table 3. Eleven studies^32–34^ ^38^ ^40^ ^42^ ^44–48^ reported CFA**;** pooled results demonstrated that PERT increased CFA versus baseline (83.7±6.0 vs 63.1±15.0; WMD: 2.28, 1.50 to 3.06; p<0.00001) with high heterogeneity (I^2^=89%). Four studies^42^ ^46–48^ reported CNA; PERT also improved CNA versus baseline (WMD: 1.01, 0.39 to 1.62; p=0.001).

**Table 3:** Results of meta-analyses for outcomes of interest

				Effect estimate		Heterogeneity	
Outcomes of interest	Studies, n	Patients, n		WMD/OR (95% CI)	p Value	I^2^ (%)	p Value
PERT vs baseline		PERT	Baseline				
CFA	11	228	229	2.28 (1.50 to 3.06)	<0.00001	89	<0.00001
CNA	4	146	147	1.01 (0.39 to 1.62)	0.001	80	0.002
FFE	13	278	281	−1.66 (−12.19 to −1.13)	<0.00001	84	<0.00001
FNE	4	93	94	−1.01 (−1.56 to −0.46)	0.0003	61	0.05
Faecal weight	6	107	111	−0.96 (−1.38 to −0.55)	<0.00001	45	0.11
Faecal consistency: *soft*	2	58	58	0.47 (0.21 to 1.06)	0.07	63	0.10
Faecal consistency: *formed/normal*	2	58	58	2.26 (1.05 to 4.89)	0.04	54	0.14
Faecal frequency	2	49	49	−0.12 (−0.52 to 0.28)	0.55	0	0.87
Flatulence	2	58	58	0.36 (0.13 to 1.02)	0.06	0	0.67
Abdominal pain	2	58	58	0.53 (0.25 to 1.12)	0.10	0	0.62
PERT vs placebo		PERT	Placebo				
CFA	7	124	114	1.67 (0.81 to 2.53)	0.0001	86	<0.00001
CNA	2	56	52	0.61 (−0.03 to 1.24)	0.06	62	0.11
FFE	7	124	114	−1.58 (−2.39 to −0.76)	0.0001	85	<0.00001
FNE	3	88	80	−0.54 (−0.85 to −0.22)	0.0007	40	0.19
Faecal weight	5	95	83	−0.92 (−1.56 to −0.28)	0.005	71	0.007
Faecal consistency: *soft*	2	58	55	0.42 (0.19 to 0.94)	0.03	0	0.89
Faecal consistency: *formed/normal*	2	58	55	1.20 (0.58 to 2.52)	0.62	87	0.006
Faecal frequency	3	70	69	−1.72 (−4.00 to 0.55)	0.14	96	<0.00001
Flatulence	2	58	55	1.20 (0.52 to 2.73)	0.67	88	0.004
Abdominal pain	2	58	55	0.37 (0.17 to 0.80)	0.01	0	0.85
Adverse event	4	104	96	1.05 (0.53 to 2.07)	0.9	0	0.83
PERT vs PERT		High dose	Low dose				
CFA	4	88	90	0.70 (−0.27 to 1.67)	0.16	69	0.02
FFE	5	103	106	−0.43 (−1.05 to 0.19)	0.18	59	0.05
Enteric-coated vs non-coated		Coated	Non-coated				
CFA	3	20	18	1.13 (−1.94 to 4.20)	0.47	91	<0.0001
FFE	4	55	53	−0.77 (−2.66 to 1.12)	0.42	89	<0.00001

CFA, coefficient of fat absorption; CNA, coefficient of nitrogen absorption; FFE, faecal fat excretion; FNE, faecal nitrogen excretion; PERT, pancreatic enzyme replacement therapy; WMD, weighted mean difference.

Thirteen papers^32–34^ ^36–38^ ^40^ ^42^ ^44–48^ reported FFE, which with FNE^32^ ^38^ ^46^ ^48^ and faecal weight^32^ ^34^ ^38^ ^42^ ^44^ ^48^ were significantly reduced by PERT (all p≤0.001). PERT improved symptoms of flatulence, abdominal pain and faecal consistency,^46^ ^48^ without significant effects on stool frequency.^37^ ^48^

### PERT versus placebo

**Figure 4:**
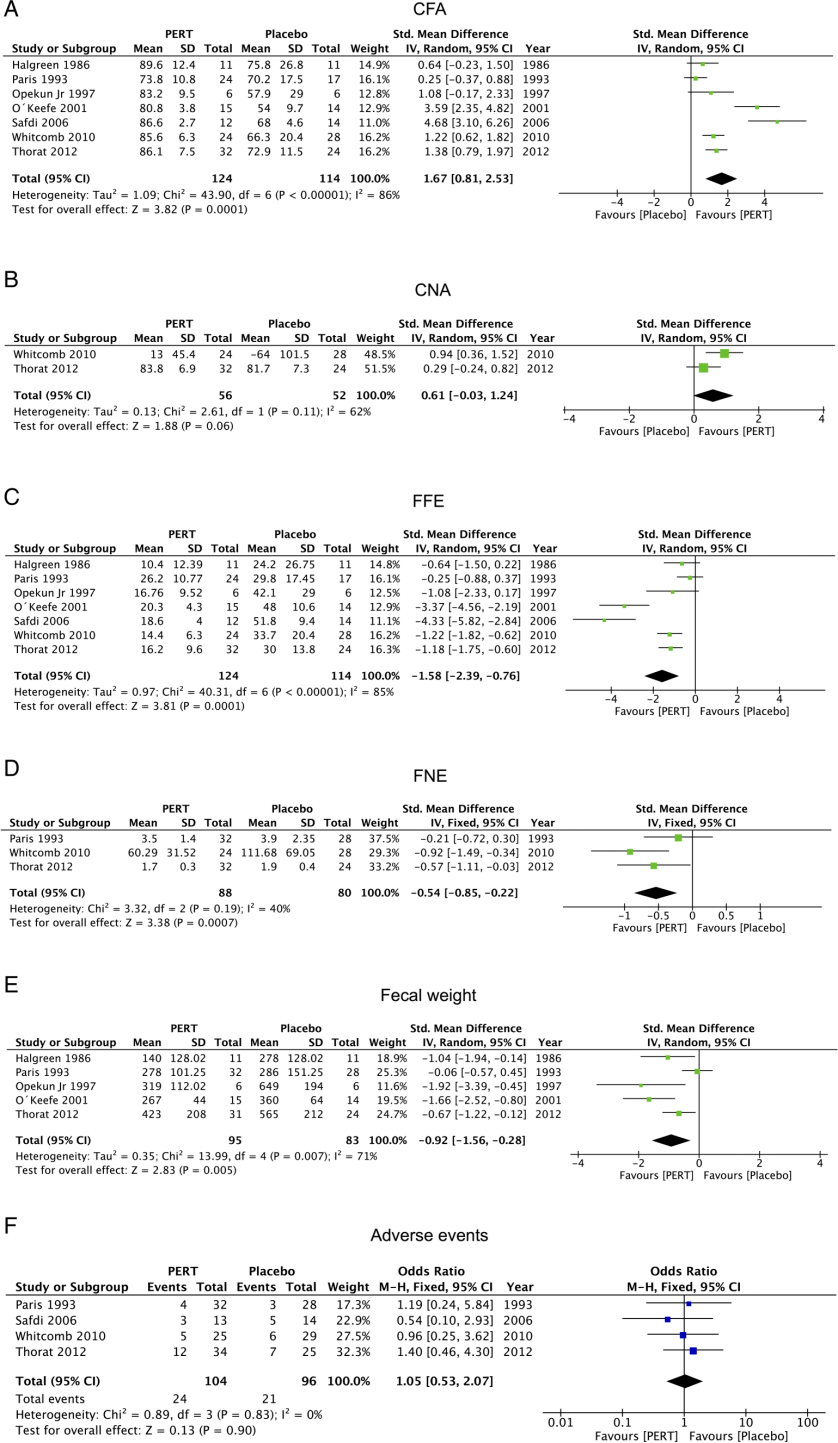
The pooled clinical outcomes of pancreatic enzyme replacement therapy (PERT) versus placebo. (A) coefficient of fat absorption (CFA), (B) coefficient of nitrogen absorption (CNA), (C) faecal fat excretion (FFE), (D) faecal nitrogen excretion (FNE), (E) faecal weight and (F) adverse events.

The clinical outcomes of PERT versus placebo are displayed in figure 4 and summarised in table 3. Pooled results from seven studies^35^ ^38^ ^40^ ^42^ ^45^ ^46^ ^48^ found that PERT greatly increased CFA over placebo (83.2±5.5 vs 67.4±7.0; WMD: 1.67, 0.81 to 2.53; p=0.0001), despite high heterogeneity (I^2^=86%). Only two^46^ ^48^ reported on CNA, showing a trend towards reduction with PERT (WMD: 0.61, −0.03 to 1.24; p=0.06).

FFE,^35^ ^38^ ^40^ ^42^ ^45^ ^46^ ^48^ FNE^38^ ^46^ ^48^ and faecal weight^35^ ^38^ ^40^ ^42^ ^48^ were consistently attenuated by PERT compared with placebo (all p≤0.005), as was abdominal pain (p=0.01),^46^ ^48^ with a tendency to improved faecal consistency but not stool frequency and flatulence.^45^ ^46^ ^48^ Pooled data from four studies^38^ ^45^ ^46^ ^48^ found adverse event profiles similar with PERT as placebo (p=0.9).

### PERT versus PERT

**Figure 5:**
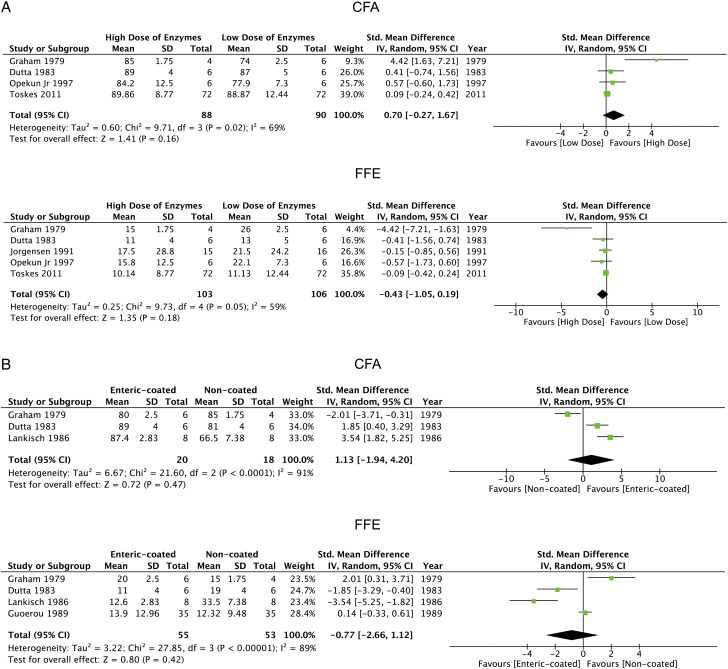
The pooled clinical outcomes of pancreatic enzyme replacement therapy (PERT) versus PERT. (A) coefficient of fat absorption (CFA) and faecal fat excretion (FFE) for high dose versus low dose and (B) CFA and FFE for enteric-coated versus non-coated.

Meta-analysis results of high versus low lipase dose regardless of delivery system are shown in figure 5A and summarised in table 3. Pooled CFA data from four studies^32^ ^33^ ^40^ ^47^ showed a higher CFA with high-dose PERT (≥60 000 USP units/day), although not statistically significant (89.2±2.0 vs 87.0±5.1; WMD: 0.70, −0.27 to 1.67; p=0.16). Pooled FFE data from these and one further study^37^ were similar, with moderate heterogeneity for CFA (I^2^=69%) and FFE (I^2^=59%). Meta-analysis of enteric-coated microspheres versus non-coated microspheres is shown in figure 5B and summarised in table 3. Pooled CFA data from three studies^32–34^ showed higher CFA with enteric-coated microspheres, although not statistically significant (85.7±4.6 vs 75.4±10.0; WMD: 0.70, −0.27 to 1.67; p=0.16). Similar findings were made from four studies^32–34^ ^36^ reporting FFE with small sample sizes (n=18–55) and high heterogeneity.

### Subgroup analysis

Subgroup analyses of PERT versus baseline for studies of high-quality, parallel multicentre, sample size ≥40 and in Western populations did not change primary meta-analysis results or statistical heterogeneity for CFA and FFE (table 4). Subgroup analyses of PERT versus placebo were similar, but heterogeneity was significantly reduced when sample size ≥40 for CFA (I^2^=86% to 74%) and FFE (I^2^=85% to 67%).

**Table 4:** Results of subgroup analyses

Subgroups	Studies, n	Patients, n	Patients, n	Effect estimate		Heterogeneity	
		PERT	Baseline or placebo	WMD/OR (95% CI)	p Value	I^2^ (%)	p Value
*PERT vs baseline*							
CFA							
High-quality studies	5	172	174	1.78 (0.85 to 2.70)	0.0002	92	<0.00001
Parallel designed	5	115	115	2.47 (1.42 to 3.52)	<0.00001	89	<0.00001
Multicentre studies	4	140	140	2.13 (0.84 to 3.43)	0.001	94	<0.00001
Sample size ≥40	4	160	160	1.26 (0.51 to 2.00)	0.001	88	<0.00001
Western population	10	196	197	1.17 (0.94 to 1.40)	<0.00001	90	<0.00001
FFE							
High-quality studies	5	172	174	−1.44 (−2.18 to −0.71)	<0.0001	88	<0.00001
Parallel designed	5	115	117	−1.66 (−2.19 to −1.13)	<0.00001	85	<0.00001
Multicentre studies	4	140	142	−1.67 (−2.67 to −0.66)	0.001	91	<0.00001
Sample size ≥40	4	160	162	−1.06 (−1.61 to −0.50)	0.0002	80	0.002
Western population	12	246	247	−1.70 (−2.29 to −1.12)	<0.00001	85	<0.00001
*PERT vs placebo*							
CFA							
High-quality studies	5	103	94	1.41 (0.51 to 2.31)	0.002	86	<0.00001
Parallel designed	5	107	97	2.03 (0.90 to 3.17)	0.0005	90	<0.00001
Multicentre studies	3	68	66	2.16 (0.85 to 3.48)	0.001	88	0.0003
Sample size ≥40	3	80	69	0.96 (0.28 to 1.64)	0.006	74	0.02
Western population	6	92	90	1.77 (0.68 to 2.86)	0.001	89	<0.00001
FFE							
High-quality studies	5	103	94	−1.32 (−2.17 to −0.47)	0.002	85	<0.0001
Parallel designed	5	107	97	−1.90 (−2.97 to −0.82)	0.0005	90	<0.00001
Multicentre studies	3	68	66	−2.00 (−3.26 to −0.75)	0.002	87	0.0004
Sample size ≥40	3	80	69	−0.89 (−1.49 to −0.29)	0.004	67	0.05
Western population	6	92	90	−1.70 (−2.74 to −0.66)	0.001	88	<0.00001

CFA, coefficient of fat absorption; FFE, faecal fat excretion; PERT, pancreatic enzyme replacement therapy; WMD, weighted mean difference.

### Sensitivity analysis

Sensitivity analyses were performed as described in online supplementary table S5. For PERT versus baseline, the results and heterogeneity of CFA and FFE were not affected by diagnostic criteria for CP or EPI or by pancreatic surgery or by only including studies with a proper run-in phase. Heterogeneity in PERT versus placebo disappeared however for both CFA (I^2^=0%; p=0.70) and FFE (I^2^=0%; p=0.92) when only including CP defined by imaging and/or histology.

10.1136/gutjnl-2016-312529.supp5 supplementary table

### Meta-regression analysis

Covariates of study design (p=0.04) and daily lipase dose (p=0.07) appeared to contribute to heterogeneity for FFE (see online supplementary table S6); age, gender, study quality and year of publication did not; limited data prevented assessment of alcohol and DM.

10.1136/gutjnl-2016-312529.supp6 supplementary table

### Publication bias

There was no significant evidence of publication bias for either CFA (see online supplementary figure S1) or FFE (see online supplementary figure S2) in PERT versus baseline, PERT versus placebo, high dose versus low dose and enteric-coated versus non-coated (Begg and Egger: p>0.10 for all comparisons).

10.1136/gutjnl-2016-312529.supp7 supplementary figures

### Extension studies

A 6-month, open-label trial^49^ (Creon 12000) extension of Whitcomb's study^46^ found PERT well tolerated, significantly improving serum nutritional parameters (retinol-binding protein, prealbumin, albumin and cholesterol) and weight, reducing faecal frequency, although no meaningful changes of QoL scores were observed. A 51-week, open-label extension trial^50^ from Thorat's study showed that Creon 40000 significantly improved laboratory nutritional parameters, fat and protein absorption, GI symptoms and QoL, with a favourable safety and tolerability profile.

## Discussion

We found PERT to improve fat and protein absorption significantly in CP, demonstrated by marked, consistent increases in CFA and CNA compared with baseline or placebo. Significant reductions in FFE, FNE, faecal weight and improvements in GI symptoms were also observed across RCTs, unchanged by subgroup, sensitivity and meta-regression analyses. Unlike in the previous Cochrane review with meta-analysis of only two studies,^21^ our meta-analysis of 14 RCTs demonstrates that PERT is clearly indicated in CP for EPI. Even though long-term effects on complications and mortality could not be determined, these findings are similar to PERT for EPI in cystic fibrosis,^22^ ^23^ which, if extrapolated, suggest long-term benefit. Although no RCT was conducted over a period longer than 2 months, two open-label extensions of up to 1 year demonstrated significant improvements in serum nutritional parameters, weight, GI symptoms and QoL. Despite PERT not wholly normalising fat absorption, driving the search for better enzymes and/or correction of other factors,^9^ the safety profile of PERT was comparable to placebo, also favourable in the extension studies.

Healthy nutrient digestion and absorption requires complex coordination of mechanical and enzymatic breakdown of food, a finely tuned process depending on integration of multiple upper GI functions including regulated, plentiful pancreatic exocrine and biliary secretion.^9^ Breakdown of fat relies on pancreatic lipase, which is highly susceptible to gastric acid; normally pancreatic bicarbonate secretion ensures intra-duodenal pH of 5–6 for optimal enzymatic activity, also preventing bile acid precipitation. One study found that endogenous lipase activity has to fall to <10% of normal before steatorrhoea occurs^15^ and thus PERT should deliver ≥10% of normal lipase activity. The amount of postprandial lipase secreted in a healthy adult is estimated at between 9000 and 18 000 USP units/min for up to 4 hours.^9^ Therefore, delivery of at least 100 000 USP units per meal (up to 400 000 USP units/120 000 international units in severe EPI) is required to correct EPI in adult CP; although we found a trend for higher doses to increase CFA, this increase was not statistically significant and did not fully correct malabsorption. Gastric acid inhibition can further enhance the efficacy of PERT;^44^ ^51^ ^52^ we found equal efficacy of PERT at higher doses alone compared with lower doses with gastric acid suppression therapy, and an RCT not varying PERT between groups found acid suppression therapy to significantly improve fat absorption.^52^ Further improvement may occur if PERT is given during meals,^43^ corresponding with normal peak enzyme secretion some 30 min after food, followed by an elevated plateau.^9^ Future RCTs are required for definitive conclusions on PERT optimisation.

EPI is frequent but variable in CP and typically progressive over a number of years,^1^ ^3^ ^5^ ^8^ contributing to long-term complications from malnutrition. Quantification of CFA and/or FFE is rarely undertaken routinely and clinical assessments of EPI are inexact; EPI may be inferred from patient and imaging characteristics, deficiencies in fat-soluble vitamins and osteoporosis, or identified by endoscopic pancreatic function testing (normal peak pancreatic bicarbonate secretion >80 mEq/L).^2^ Lipid-soluble vitamins, retinol-binding protein, albumin and prealbumin may be useful to monitor responses to PERT.^49^ ^50^ Nevertheless, once the diagnosis of CP is established and since some degree of EPI is likely,^3^ ^8^ ^16–18^ PERT is the treatment of choice to reduce and/or minimise long-term malnutrition, unless pancreatic secretion is demonstrated to be normal. Support for this comes from a prospective, non-randomised, multicentre 1-year cohort study of 206 patients with EPI from CP already on PERT and 88 with newly diagnosed EPI from CP newly prescribed PERT.^53^ EPI was identified by maldigestion, diarrhoea/steatorrhoea, weight loss, meteorism, dyspepsia, recurrent pain, nausea and vomiting. PERT was associated with significant reductions in recurrent abdominal pain, GI symptoms and GI QoL index (all p<0.001) in both cohorts, although uncertainty remains as to how PERT reduces intestinal and/or pancreatic pain.

Despite use of the more conservative random-effects model, we found significant heterogeneity between studies. Subgroup analyses, however, did not alter estimates of the effect of PERT versus baseline or placebo on CFA and FFE, nor estimates of heterogeneity, although restriction of analysis to larger studies reduced heterogeneity. Sensitivity analyses did not alter estimates for CFA and FFE, but heterogeneity was abolished for FFE in PERT versus placebo when CP was diagnosed by imaging and/or histology criteria. The heterogeneity identified highlights the need for greater international consensus on the definition and diagnosis of CP.^54^

Access to medical expertise, compliance, diet and lifestyle is heavily influenced by health inequalities.^4^ Proxy indicators, notably alcohol usage and cigarette smoking, are themselves independently associated with progression of CP and nutrient deprivation.^4^ In our study, 76.4% of patients were male and 89.1% of patients had alcohol-associated CP, but none of the RCTs reported measures of smoking, residence, socioeconomic or employment status, diet or comorbidity. As genetic factors and smoking have become increasingly recognised in CP progression,^2^ ^3^ ^4^ health inequalities should be addressed in future studies of PERT to increase the applicability of findings to all patients with CP.
