# Supplementary material for: Efficacy of pancreatic enzyme replacement therapy in chronic pancreatitis: systematic review and meta-analysis
Source: Gut. 2016 Dec 9;66(8):1354–5. doi: 10.1136/gutjnl-2016-312529 (PMC5530474; doi:10.1136/gutjnl-2016-312529)
Supplement: supplementary table [file gutjnl-2016-312529supp001.pdf]

Table S1. Detailed Jadad score components*

Study	Jadad score	Randomization	Double blinding	Drop-outs or withdrawals	Allocation concealment
Graham[32]	1	1	0	0	N
Dutta et al[33]	1	1	0	0	N
Lankish et al[34]	1	1	0	0	N
Halgreen et al[35]	3	1	2	0	N
Gouerou et al[36]	2	1	0	1	N
Jorgensen et al[37]	1	1	0	0	N
Paris et al[38]	3	1	1	1	N
Delhaye et al[39]	2	1	0	1	N
Opekun Jr et al[40]	1	1	0	0	N
Halm et al[41]	4	1	2	1	N
O'Keefe et al[42]	2	1	0	1	N
Domínguez-Muñoz et al[43]	2	2	0	0	Y
Vecht et al[44]	2	1	1	0	N
Safdi et al[45]	3	1	1	1	N
Whitcomb et al[46]	5	2	2	1	Y
Toskes et al[47]	3	1	1	1	N
Thorat et al[48]	5	2	2	1	Y

Y, reported; N, not reported.

*One point if study described as randomized, further point if method described and appropriate but one point deducted if method described and inappropriate; one point if study described as double blind, further point if method described and appropriate but one point deducted if method described and inappropriate; one point if withdrawals and/or drop outs described with reasons; maximum total 5 points.[25]
