# Supplementary material for: Efficacy of pancreatic enzyme replacement therapy in chronic pancreatitis: systematic review and meta-analysis
Source: Gut. 2016 Dec 9;66(8):1354–5. doi: 10.1136/gutjnl-2016-312529 (PMC5530474; doi:10.1136/gutjnl-2016-312529)
Supplement: supplementary table [file gutjnl-2016-312529supp002.pdf]

**Table S2.** Baseline characteristics of patients in included studies*

| Study | No. patients (analysed) | Age (year)† | Male/ Female | Ethnicity | Body mass index (kg/m$^2$)† | No. with coexisting diabetes mellitus | No. after pancreatic surgery | Aetiology |
|---|---|---|---|---|---|---|---|---|
| Graham[32] | 6 (6) | 44-59 | 4/2 | NR | NR | 0 | 0 | Alcoholic 6 |
| Dutta el al[33] | 6 (6) | 50 (43-58) | NR | NR | NR | 3 | Yes, number not given | Alcoholic 6 |
| Lankisch et al[34] | 8 (8) | NR | 7/1 | NR | NR | NR | Yes, number not given | Alcoholic 7<br>Idiopathic 1 |
| Halgreen et al[35] | 11 (11) | 51 (29-59) | 6/5 | NR | NR | 6 | 0 | Alcoholic 4; Idiopathic 3; Obstructive 3; Hyperlipidemic 1 |
| Gouerou et al[36] | 35 (23) | 50 (18-75) | 33/2 | NR | NR | 16 | 25, type unknown | Alcoholic 33<br>Idiopathic 2 |
| Jorgensen et al[37] | 23 (23) | 51 (34-73) | 18/5 | NR | NR | NR | 0 | NR |
| Paris et al[38] | 60 (60) | 47 | 51/9 | NR | NR | NR | Yes, number not given | Alcoholic 60 |
| Delhaye et al[39] | 25 (25) | 52.4 (1.7) | 24/1 | NR | NR | 16 | 9‡ | Alcoholic 23 Idiopathic 2 |
| Opekun Jr et al[40] | 6 (6) | 37-63 | NR | NR | NR | 2 | 0 | Alcoholic 5; Idiopathic 1 |
| Halm et al[41] | 37 (23) | 52 (10) | 30/7 | NR | 19 (3) | NR | 0 | NR |
| O´Keefe et al[42] | 29 (29) | G1: 49.1 (1.8)<br>G2: 57.8 (2.1) | 28/1 | NR | G1: 18.9 (0.7)<br>G2: 22.3 (1.0) | 18 | 9§ | Alcoholic 27; Idiopathic 2 |
| Domínguez-Muñoz et al[43] | 24 (24) | 50 (36-76) | 19/5 | NR | NR | No | 0 | NR |
| Vecht et al[44] | 16 (16) | 53 (3) | 13/3 | NR | NR | 6 | 4‖ | Alcoholic 9; Obstructive 1; Idiopathic 6 |
| Safdi et al[45] | 27 (27) | G1: 51.9 (2.7)<br>G2: 51 (3) | 9/18 | White 16<br>AA 10<br>Filipino 1 | NR | NR | 0 | NR |
| Whitcomb et al[46] | 54 (54)¶ | G1: 52 (9.6)<br>G2: 50.5 (7.7) | 39/15 | White 53<br>Black 1 | G1: 23.4 (4.4)<br>G2: 22.2 (4.4) | NR | 14, type unknown | NR |
| Toskes et al[47] | 82 (75) | 51.9 (12.1) | 53/29 | White 17<br>Black 2<br>Hispanic 2 | 23.4 (4.4) | NR | Yes** | NR |
| Thorat et al[48] | 62 (61) | 44 (18-62) | 47/15 | Asian 62 | G1: 19.1 (3.1)<br>G2: 18.4 (2.5) | 30 | 18, type unknown | NR |

NR, not reported; G, group; AA, African American.

[*]Socioeconomic status and cigarette smoking not reported in any study included.

[†]Expressed either as mean, mean (standard deviation), range, or median (range).

[‡]Left pancreatectomy, 6; pancreatico-jejunostomy, 2; and cystogastromy, 1.

[§]Pancreatic decompression surgery, 9.

[||]Pancreatic drainage, 2; duodenum preserving resection of the head of the pancreas, 2.

[¶]One patient who had pancreatic cancer resection was unavoidably included in the analysis.

[**]Partial or distal pancreatic resection.
