# Supplementary material for: Efficacy of pancreatic enzyme replacement therapy in chronic pancreatitis: systematic review and meta-analysis
Source: Gut. 2016 Dec 9;66(8):1354–5. doi: 10.1136/gutjnl-2016-312529 (PMC5530474; doi:10.1136/gutjnl-2016-312529)
Supplement: supplementary table [file gutjnl-2016-312529supp003.pdf]

Table S3. Composition and administration schedules of PERT

Study	Formulation per capsule (lipase/other enzymes)	Formulation per capsule (enzyme activity in USP)[*]	Delivery system[†]	Doses and administration schedule[‡]	Antacids[§]
Graham[32]	Ilozyme (U)<br>*Lipase: 3600*<br><br>Pancrease (U)<br>*Lipase: 2005*	The same as original	Ilozyme<br>*Non-EC, MT*<br><br>Pancrease<br>*EC, MS*	Ilozyme<br>*Meal: 3/6/12 tablets tid*<br><br>Pancrease<br>*Meal: 3 capsules tid*	NR
Dutta el al[33]	Pancreatin (U)<br>*Lipase: 684 ± 52, protease: 795 ± 19*<br><br>Cotazyme (U)<br>*Lipase: 8804 ± 159, protease: 9937 ± 243*<br><br>Pancrease TM (U)<br>*Lipase: 4933 ± 64, protease: 6183 ± 363*	Pancreatin<br>*Lipase: 684, protease: 795*<br><br>Cotazyme<br>*Lipase: 8804, protease: 9937*<br><br>Pancrease TM<br>*Lipase: 4933, protease: 6183*	Pancreatin:<br>*Non-EC, MS*<br><br>Cotazyme:<br>*Non-EC, MT*<br><br>Pancrease TM<br>*EC, MT*	Pancreatin<br>*Meal: 10 tablets tid*<br><br>Cotazyme<br>*Meal: 4 capsules tid*<br><br>Pancrease TM<br>*Meal: 4/8 capsules tid*	NR
Lankisch et al[34]	Pankreon® 700 (FIP)<br>*Lipase: 28000, protease: 1500, amylase: 22000*<br><br>Kreon®<br>*Lipase: 10000*	Pankreon® 700<br>*Lipase: 28000, protease: 93750, amylase: 91300*<br><br>Kreon®<br>*Lipase:10000*	Pankreon® 700<br>*Non-EC, MT*<br><br>Kreon®<br>*EC, granules*	Pankreon® 700<br>*Meal: 3 doses tid*<br><br>Kreon®<br>*Meal: 6 capsules tid*	Yes
Halgreen et al[35]	Pancrease® (NFU)<br>*Lipase: 4000, protease: 25000, amylase: 20000*	NA	Pancrease®<br>*EC, MS*	*Pancrease®*<br>*Meal: 2 capsules tid; snack: 1 capsule bid*	NR
Gouerou et al[36]	NA	NA	Alipase®<br>*EC, MS*<br><br>Eurobiol<br>*Non-EC*	Alipase®<br>*Meal: 9 capsules qd*<br><br>Eurobiol<br>*Meal: 3 vials qd*	NR
Jorgensen et al[37]	Pancrease® (EPU)<br>*Lipase: 5000, protease: 330, amylase: 2900*<br><br>Pankreon® (EPU)<br>*Lipase: 8000, protease: 450, amylase: 9000*<br><br>Pankreatin® (EPU)<br>*Lipase: 180000, protease: 7975, amylase: 191250*	Pancrease®<br>*Lipase: 5000, protease: 20625, amylase: 12035*<br><br>Pankreon®<br>*Lipase: 8000, protease: 28125, amylase: 37350*<br><br>Pankreatin®<br>*Lipase: 180000, protease: 498437, amylase: 793791*	Pancrease®<br>*EC, MS*<br><br>Pankreon®<br>*EC, MS*<br><br>Pankreatin®<br>*EC, granules*	Pancrease®<br>*Meal: 2 capsules tid; snack: 1 capsule bid*<br><br>Pankreon®<br>*Meal: 2 capsules tid; snack: 1 capsule bid*<br><br>Pankreatin®<br>*Meal: 15 ml tid; snack: 7.5 ml bid*	No
Paris et al[38]	Panzytrat (EPU)<br>*Lipase: 25000, protease: 1250, amylase: 22500*	Panzytrat<br>*Lipase: 25000, protease: 78125, amylase: 93375*	Panzytrat<br>*EC, MT*	Panzytrat<br>*Meals: 6 capsules tid*	Not allowed
Delhaye et al[39]	Pancrease HL (EPU)<br>*Lipase: 25000, protease: 1250, amylase 22500*<br><br>Creon<br>*Lipase: 8000, protease: 450, amylase: 9000*	Pancrease HL<br>*Lipase: 25000, protease: 78125, amylase 93375*<br><br>Creon<br>*Lipase: 8000, protease: 28125, amylase: 37350*	Pancrease HL<br>*EC, MS*<br><br>Creon<br>*EC, MS*	Pancrease HL<br>*Meal: 1 capsule tid*<br><br>Creon<br>*Meal: 3 capsule tid*	Yes
Opekun et al[40]	Pancrease MT4 (USP)<br>*Lipase: 4000, protease: 12000, amylase: 12000*<br><br>Pancrease MT10 (USP)<br>*Lipase: 10000, protease: 30000, amylase: 30000*<br>Pancrease MT16<br>*Lipase: 16000, protease: 48000, amylase: 48000*	The same as original	Pancrease MT4/MT10/MT16<br>*EC, MT*	Pancrease MT4<br>*Meal: 2 capsules tid (before)*<br><br>Pancrease MT10<br>*Meal: 2 capsules tid (before)*<br>Pancrease MT16<br>*Meal: 2 capsules tid (before)*	Not allowed

Halm et al[41]	Creon 10000 MS (EPU)<br>*Lipase: 10000, protease: 600, amylase: 8000*<br><br>Creon 10000 MMS (EPU)<br>*Lipase: 10000, Protease: 600, Amylase: 8000*	Creon 10000 MS<br>*Lipase: 10000, protease: 37500, amylase: 33200*<br><br>Creon 10000 MMS<br>*Lipase: 10000, protease: 37500, amylase: 33200*	Creon 10000 MS<br>*EC, MS*<br>Creon 10000 MMS<br>*EC, MMS*	Creon 10000 MS<br>*Meal: 4 capsules tid; snack: 2 capsules bid*<br><br>Creon 10000 MMS<br>*Meal: 4 capsules tid; snack: 2 capsules bid*	Not allowed
O´Keefe et al[42]	Creon 10 (USP)<br>*Lipase: 10000, protease: 37500, amylase: 33200*	The same as original	Creon 10<br>*EC, MMS*	Creon<br>*Meal: 4 capsules tid; snack: 2 capsules bid*	Not allowed
Domínguez-Muñoz et al[43]	Creon 10000 (EPU)<br>*Lipase: 10000, protease: 600, amylase: 8000*	Creon 10000<br>*Lipase: 10000, protease: 37500, amylase: 33200*	Creon 10000<br>*EC, MMS*	Creon 10000<br>*Meal and snack: 4 capsules tid (before, after or during)*	Not allowed
Vecht et al[44]	Pancrease (FIP)<br>*Lipase: 5000, protease: 330, amylase: 2900*	Pancrease<br>*Lipase: 5000, protease: 20625, amylase: 12035*	Pancrease<br>*EC, MS*	Pancrease<br>*Meal and snack: 2 or 4 capsules tid for low or high does, respectively (before)*	Yes
Safdi et al[45]	Creon 10 (USP)<br>*Lipase: 10000, protease: 37500, amylase: 33200*	The same as original	Creon 10<br>*EC, MMS*	Creon 10<br>*Meal: 4 capsules tid; snack: 2 capsules bid*	Not allowed
Whitcomb et al[46]	Creon 12000 (USP)<br>*Lipase: 12000, protease: 38000, amylase: 60000*	The same as original	Creon 12000<br>*EC, MMS*<br>*Delayed-release*	Creon 12000<br>*Meal: 6 capsules tid; snack: 3 capsules bid*	Allowed
Toskes et al[47]	Zenpep 5000 (USP)<br>*Lipase: 5000, protease: 17000, amylase: 27000*<br><br>Zenzep 20000 (USP)<br>*Lipase: 20000, protease: 68000, amylase: 109000*	The same as original	*Zenpep 5000*<br>*EC, MS*<br>*Delayed-release*<br><br>*Zenpep 20000*<br>*EC, MS*<br>*Delayed-release*	Zenpep 5000<br>*Meal: 2 capsules tid; snack: 1 capsule bid*<br><br>Zenpep 2000<br>*Meal: 2 capsules tid; snack: 1 capsule bid*	Not allowed
Thorat et al[48]	Creon 40000 (EPU)<br>*Lipase: 40000, protease: 1600, amylase: 25000*	Creon 40000<br>*Lipase: 40000, protease: 100000, amylase: 103750*	Creon 40000<br>*EC, MMS*	Creon 40000<br>*Meal: 2 capsules tid; snack: 1 capsule bid*	Allowed

USP, United States Pharmacopoeia; NR, not reported; MT, microtablets; NA, not available; MS, microspheres; MMS, minimicrospheres; EC, enteric-coated; Non-EC, non-coated; FIP, Federation Internationale Pharmaceutique; NFU, National Formulary Unit; EPU, European Pharmacopoeia Unit.

*Conversion factor is shown in Table S4.

†Granules/MT/MS/MMS and EC

‡Administered with meals and/or snacks if not otherwise mentioned.

§Antisecretory drugs
