# Supplementary material for: Efficacy of pancreatic enzyme replacement therapy in chronic pancreatitis: systematic review and meta-analysis
Source: Gut. 2016 Dec 9;66(8):1354–5. doi: 10.1136/gutjnl-2016-312529 (PMC5530474; doi:10.1136/gutjnl-2016-312529)
Supplement: supplementary table [file gutjnl-2016-312529supp004.pdf]

Table S4. Source of pancreatic enzyme supplements and activity conversion factors

Study	Name and origin of enzyme	Lipase, protease and amylase conversion factor*
Graham[32]	Ilozyme<br>*Warren-Teed Pharmaceuticals, USA*<br><br>Pancrease<br>*Janssen-Cilag (Johnson & Johnson), USA*	Ilozyme<br>*Lipase: 0.36*<br><br>Pancrease<br>*Lipase: 0.2*
Dutta el al[33]	Pancreatin<br>*Eli Lilly & Co., USA*<br><br>Cotazyme<br>*Organon Inc., USA*<br><br>Pancrease TM<br>*Janssen-Cilag (Johnson & Johnson), USA*	Pancreatin<br>*Lipase: 0.0684, protease: 0.02*<br><br>Cotazyme<br>*Lipase: 0.88, protease: 0.26*<br><br>Pancrease TM<br>*Lipase: 0.49, protease: 0.16*
Lankisch et al[34]	Pankreon® 700<br>*Kali-Chemie Pharme, Germany*<br><br>Kreon®<br>*Kali-Chemie Pharme, Germany*	Pankreon® 700<br>*Lipase: 2.8, protease: 2.5, amylase: 2.75*<br><br>Kreon®<br>*Lipase:1*
Halgreen et al[35]	Pancrease®<br>*Janssen-Cilag (Johnson & Johnson), Sweden*	NA
Gouerou et al[36]	Alipase®<br>*Janssen-Cilag (Johnson & Johnson), France*<br><br>Eurobiol<br>*Mayoly Spindler, France*	NA
Jorgensen et al[37]	Pancrease®<br>*Janssen-Cilag (Johnson & Johnson), Sweden*<br><br>Pankreon®<br>*Kalichemie, Germany*<br><br>Pankreatin®<br>*Rosco, Denmark*	Pancrease®<br>*Lipase: 0.5, protease: 0.55, amylase: 0.36*<br><br>Pankreon®<br>*Lipase: 0.8, protease: 0.75, amylase: 1.12*<br><br>Pankreatin®<br>*Lipase: 18, protease: 13.29, amylase: 23.91*
Paris et al[38]	Panzytrat<br>*Knoll BASF Pharma, Germany*	Panzytrat<br>*Lipase: 2.5, protease: 2.08, amylase: 2.81*

Delhaye et al[39]	Pancrease HL<br>*Janssen-Cilag (Johnson & Johnson), Belgium*<br><br>Creon<br>*Solvay Pharmaceuticals, Germany*	Pancrease HL<br>*Lipase: 2.5, protease: 2.08, amylase 2.81*<br><br>Creon<br>*Lipase: 0.8, protease: 0.75, amylase: 1.12*
Opekun et al[40]	Pancrease MT4/MT10/MT16<br>*McNeil, Consumer Products Co., USA*	Pancrease MT4<br>*Lipase: 0.4, protease: 0.32, amylase: 0.36*<br><br>Pancrease MT10<br>*Lipase: 1, protease: 0.8, amylase: 0.9*<br><br>Pancrease MT16<br>*Lipase: 1.6, protease: 1.28, amylase: 1.45*
Halm et al[41]	Creon 10000 MS/10000 MMS<br>*Solvay Pharmaceuticals, Germany*	Creon 10000 MS/10000 MMS<br>*Lipase: 1, protease: 1, amylase: 1*
O´Keefe et al[42]	Creon 10<br>*Solvay Pharmaceuticals, USA*	Creon 10<br>*Lipase: 1, protease: 1, amylase: 1*
Domínguez-Muñoz et al[43]	Creon 10000<br>*Solvay Pharmaceuticals, Germany*	Creon 10000<br>*Lipase: 1, protease: 1, amylase: 1*
Vecht et al[44]	Pancrease<br>*Janssen-Cilag (Johnson & Johnson), Netherlands*	Pancrease<br>*Lipase: 0.5, protease: 0.55, amylase: 0.36*
Safdi et al[45]	Creon 10<br>*Solvay Pharmaceuticals, USA*	Creon 10<br>*Lipase: 1, protease: 1, amylase: 1*
Whitcomb et al[46]	Creon 12000<br>*Solvay Pharmaceuticals., USA*	Creon 12000<br>*Lipase: 1.2, protease: 1.01, amylase: 1.81*
Toskes et al[47]	EUR-1008 (Zenpep)<br>*Eurand Pharmaceuticals Inc., USA*	Zenpep 5000<br>*Lipase: 0.5, protease: 0.45, amylase: 0.81*<br><br>Zenzep 20000<br>*Lipase: 2, protease: 1.81, amylase: 3.28*
Thorat et al[48]	Creon 40000<br>*Abbott Pharmaceutical, Germany*	Creon 40000<br>*Lipase: 4, protease: 2.67, amylase: 3.13*

NA, not available.

*Conversion factor is based on current dose of enzymes in Kreon 10000.
