# Supplementary material for: Efficacy of pancreatic enzyme replacement therapy in chronic pancreatitis: systematic review and meta-analysis
Source: Gut. 2016 Dec 9;66(8):1354–5. doi: 10.1136/gutjnl-2016-312529 (PMC5530474; doi:10.1136/gutjnl-2016-312529)
Supplement: supplementary table [file gutjnl-2016-312529supp005.pdf]

Table S5. Results of sensitivity analyses

	No. studies	No. patients		Effect estimate		Heterogeneity	
				**WMD/OR (95%CI)**	***P* value**	**I^2 (%)**	***P* value**
**PERT vs baseline**							
**CFA:**		**PERT**	**Baseline**				
CP defined by imaging and/or histology	4	134	134	0.99 (0.72 to 1.25)	<0.00001	90	<0.00001
CP defined by imaging and/or clinical history	3	63	63	1.65 (1.21 to 2.09)	<0.00001	93	<0.00001
EPI defined by FFE >7-8 g/d	1	6	6	NA	NA	NA	NA
EPI defined by CFA ≤80% and/or FFE >10-15 g/d	7	139	139	2.50 (1.59 to 3.40)	<0.00001	87	<0.00001
Studies including patients after pancreatic surgery	8	205	205	1.96 (1.18 to 2.75)	<0.00001	89	<0.00001
Studies with proper run-in phase	9	217	217	2.08 (1.28 to 2.88)	<0.00001	90	<0.00001
**FFE:**		**PERT**	**Baseline**				
CP defined by imaging and/or histology	5	169	171	-1.13 (-1.73 to -0.53)	0.0002	81	.0003
CP defined by imaging and/or clinical history	4	78	78	-1.82 (-2.92 to -0.72)	0.001	87	<0.0001
EPI defined by FFE >7-8 g/d	2	41	41	-1.57 (-3.85 to 0.71)	0.18	83	0.01
EPI defined by CFA ≤80% and/or FFE >10-15 g/d	8	154	156	-1.85 (-2.48 to -1.22)	<0.00001	80	<0.0001
Studies including patients after pancreatic surgery	9	240	242	-1.50 (-2.05 to -0.94)	<0.00001	84	<0.00001
Studies with proper run-in phase	11	267	269	-1.49 (-2.01 to -0.97)	<0.00001	84	<0.00001
**PERT vs placebo**							
**CFA:**		**PERT**	**Placebo**				
CP defined by imaging and/or histology	2	56	52	1.30 (0.88 to 1.72)	<0.00001	0	0.70
CP defined by imaging and/or clinical history	3	50	42	0.85 (0.38 to 1.31)	0.0004	91	<0.0001
EPI defined by FFE >7-8 g/d	0	NA	NA	NA	NA	NA	NA
EPI defined by CFA ≤80% and/or FFE >10-15 g/d	5	107	97	2.03 (0.90 to 3.17)	0.0005	90	<0.00001
Studies including patients after pancreatic surgery	4	95	83	1.49 (0.50 to 2.47)	0.003	87	<0.0001
Studies with proper run-in phase	7	124	114	1.67 (0.81 to 2.53)	0.0001	86	<0.00001
**FFE:**		**PERT**	**Placebo**				
CP defined by imaging and/or histology	2	56	52	-1.20 (-1.61 to -0.78)	<0.00001	0	.92
CP defined by imaging and/or clinical history	3	50	42	-1.35 (-2.98 to 0.27)	0.10	91	<0.0001
EPI defined by FFE >7-8 g/d	0	NA	NA	NA	NA	NA	NA
EPI defined by CFA ≤80% and/or FFE >10-15 g/d	5	107	97	-1.90 (-2.97 to -0.82)	0.0005	90	<0.00001
Studies including patients after pancreatic surgery	4	95	83	-1.39 (-2.31 to -0.46)	0.003	86	<0.0001
Studies with proper run-in phase	7	124	114	-1.58 (-2.39 to -0.76)	0.0001	85	<0.00001

PERT, pancreatic enzyme replacement therapy; WMD, weighted mean difference; OR, odds ratio; CI, confidence interval; CFA, coefficient of fat absorption; CP, chronic pancreatitis; EPI, exocrine pancreatic insufficiency; FFE, faecal fat excretion; NA, not available.
