# Supplementary material for: Efficacy of pancreatic enzyme replacement therapy in chronic pancreatitis: systematic review and meta-analysis
Source: Gut. 2016 Dec 9;66(8):1354–5. doi: 10.1136/gutjnl-2016-312529 (PMC5530474; doi:10.1136/gutjnl-2016-312529)
Supplement: supplementary table [file gutjnl-2016-312529supp006.pdf]

Table S6. Results of meta-regression analyses

		Coefficient (95%CI), *P* value[*]						
		**Year of publication**	**Gender (men, %)**	**Age (year)**	**Study design**	**Study quality**	**Lipase dose/d**	**Adj R-squared[†] (%)**
**Each covariate fitted into individual model**	**PERT vs baseline**							
	CFA	-0.46 (1.15 to 0.23), 0.17	-0.08 (-0.69 to 0.53), 0.76	1.48 (-1.83 to 4.65), 0.35	-1.92 (-19.57 to 15.73), 0.81	-9.84 (-27.78 to 8.09), 0.25	-0.00 (-0.0001 to 0.00), 0.38	
	FFE	0.21 (-0.68 to 1.09), 0.62	0.49 (-0.11 to 1.1), 0.09	-0.90 (-5.06 to 3.26), 0.64	-9.37 (-28.47 to 9.72), 0.3	5.28 (-15.14 to 25.71), 0.58	0.00 (-0.00002 to 0.0008), 0.27	
	**PERT vs placebo**							
	CFA	0.18 (-0.83 to 1.2), 0.66	0.01 (-0.46 to 0.49), 0.94	1.13 (-1.63 to 3.89), 0.34	-4.29 (-29.61 to 21.02), 0.68	-15.31 (-51.69 to 21.07), 0.33	-0.00002 (-0.0001 to 0.0001), 0.6	
	FFE	-0.36 (-1.59 to 0.87), 0.49	0.19 (-0.4 to 0.79), 0.41	-1.94 (-5.31 to 1.42), 0.19	-1.33 (-30.97 to 28.31), 0.91	6.10 (-36.82 to 49. 02), 0.73	0.00003 (-0.0001 to 0.0002), 0.59	
**Each covariate fitted into multivariable model**	**PERT vs baseline**							
	CFA	1.36 (-18.97 to 21.68), 0.55	0.08 (-3.46 to 3.63), 0.82	0.98 (-25.22 to 27.19), 0.72	33.54 (-232.98 to 300.07), 0.36	-74.81 (-747.41 to 597.78), 0.39	-0.00 (-0.001 to 0.001), 0.46	23.2
	FFE	-0.46 (-3.17 to 2.24), 0.54	0.29 (-0.33 to 0.93), 0.18	-1.53 (-5.48 to 2.43), 0.24	-34.69 (-66.27 to -3.12), 0.04	52.25 (-39.89 to 144.39), 0.14	0.00 (-0.0001 to 0.00016), 0.07	94.8
	**PERT vs placebo**							
	CFA[‡]	2.66 (-6.75 to 12.07), 0.17	0.34 (-1.02 to 1.7), 0.19				-0.0002 (-0.0009 to 0.0005), 0.17	90.0
	FFE[§]	-3.11 (-11.46 to 5.23), 0.13	-0.16 (-1.35 to 1.04), 0.34				0.0003 (-0.0004 to 0.0009), 0.12	100

CI, confidence interval; PERT, pancreatic enzyme replacement therapy; CFA, coefficient of fat absorption; FFE, faecal fat excretion.

[*]Two-sided *P* value.

[†]Proportion of variability among studies explained.

[‡,§]Included covariates in the full model which have more influence in the heterogeneity.
