# Supplementary material for: Efficacy of pancreatic enzyme replacement therapy in chronic pancreatitis: systematic review and meta-analysis
Source: Gut. 2016 Dec 9;66(8):1354–5. doi: 10.1136/gutjnl-2016-312529 (PMC5530474; doi:10.1136/gutjnl-2016-312529)
Supplement: supplementary figures [file gutjnl-2016-312529supp007.pdf]

Supplementary Figure Legends

Figure S1. Funnel plots of publication bias for CFA. (*A*) PERT vs baseline (Begg: p=0.46, Egger: p=0.632), (B) PERT vs placebo (Begg: p=0.46, Egger: p=0.69) and PERT vs PERT including (C) High dose vs low dose (Begg: p=0.29, Egger: p=0.101) and (D) Enteric-coated vs non-coated (Begg: p=0.29, Egger: p=0.101). CFA, coefficient of fat absorption.

Figure S2. Funnel plots of publications bias for FFE. (A) PERT vs baseline (Begg: p=0.71, Egger: p=0.16), (B) PERT vs placebo (Begg: p=0.29; Egger: p=0.36), and PERT vs PERT including (C) High dose vs low dose (Begg: p=1, Egger: p=0.58) and (D) Enteric-coated vs non-coated (Begg: p=0.31, Egger: p=0.22). FFE, fecal fat excretion.

Figure S1

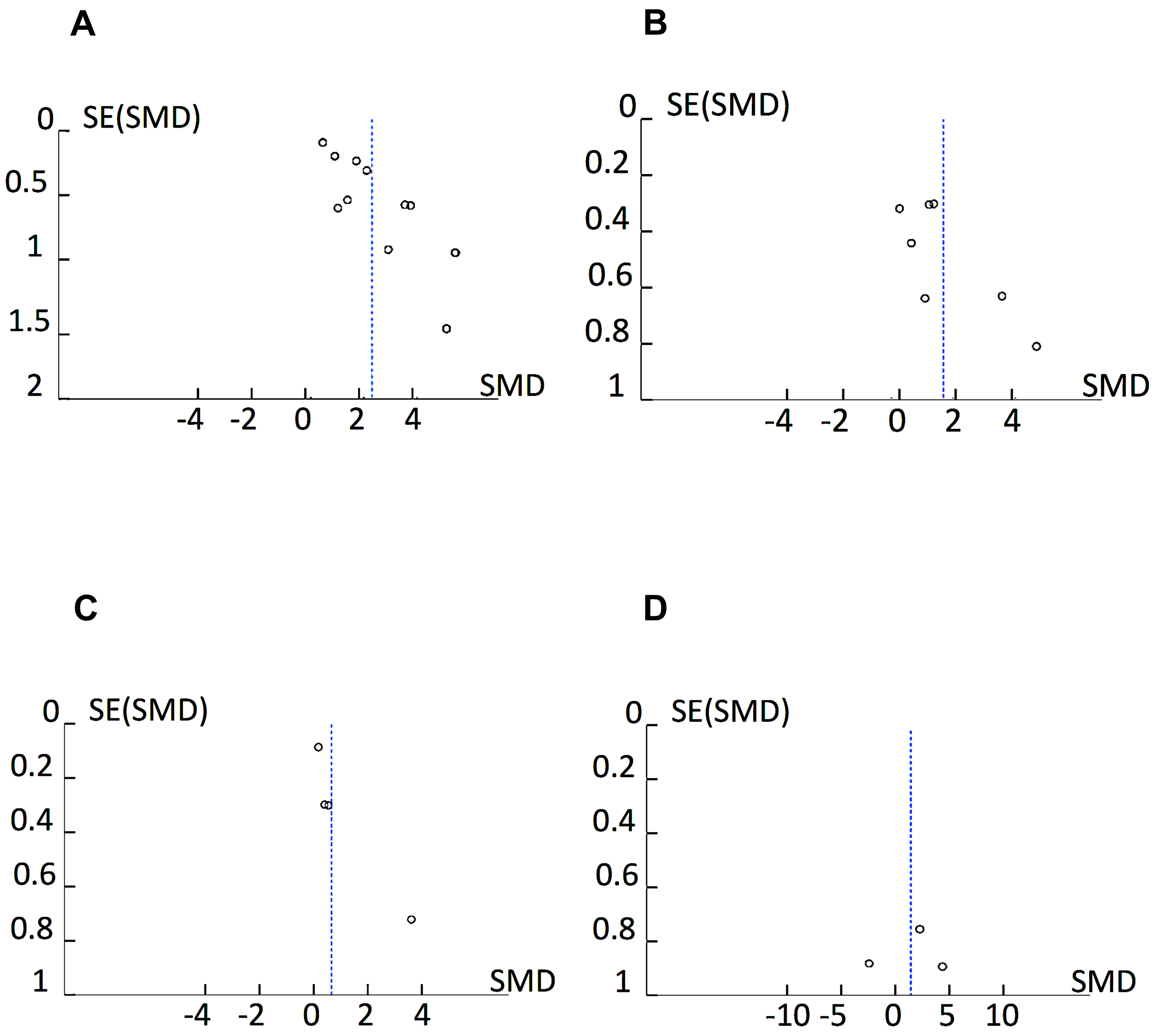

Figure S2

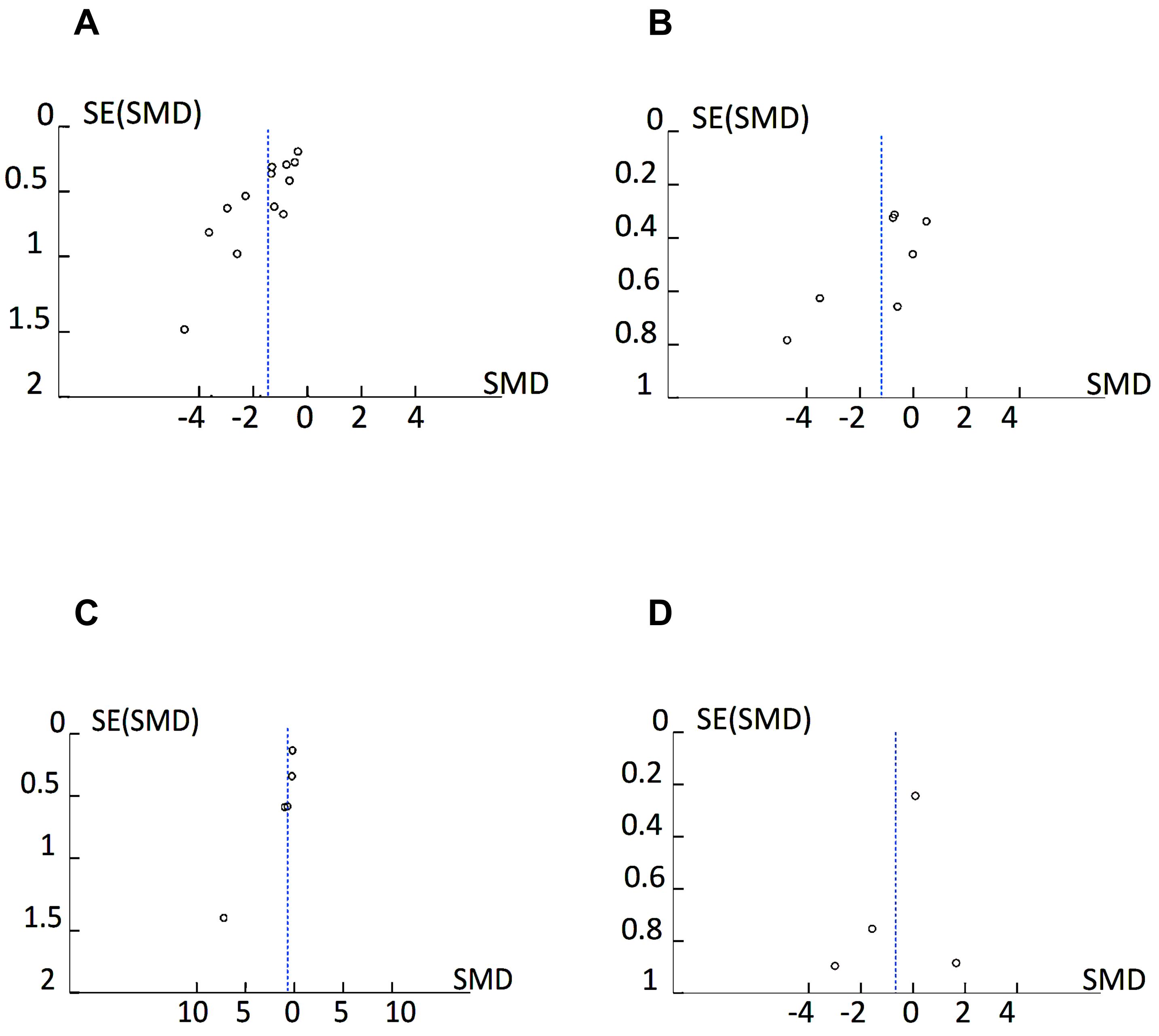

A
SE(SMD)
0
0.5
1
1.5
2
SMD
-4 -2 0 2 4
B
SE(SMD)
0
0.2
0.4
0.6
0.8
1
SMD
-4 -2 0 2 4
C
SE(SMD)
0
0.5
1
1.5
2
SMD
10 5 0 5 10
D
SE(SMD)
0
0.2
0.4
0.6
0.8
1
SMD
-4 -2 0 2 4
